# Strength Prediction of Ball-Milling-Modified Phosphorus Building Gypsum Based on NSGM (1,4) Model

**DOI:** 10.3390/ma15227988

**Published:** 2022-11-11

**Authors:** Yi Zhang, Zhong Tao, Lei Wu, Zhiqi Zhang, Zhiman Zhao

**Affiliations:** 1 Faculty of Civil Engineering and Mechanics, Kunming University of Science and Technology, Kunming 650500, China; 2 Yunnan Earthquake Engineering Research Institute, Kunming 650500, China; 3 Yunnan Ningchuang Environmental Technology Co., Ltd., Anning 650300, China

**Keywords:** phosphorus building gypsum, ball milling, strength prediction, NSGM (1,4)

## Abstract

Phosphogypsum is an industrial byproduct from the wet preparation of phosphoric acid. Phosphorus building gypsum can be obtained from phosphogypsum after high-thermal dehydration. This study aimed to analyze the influence of ball milling with different parameters on the strength of phosphorus building gypsum. In this paper, the absolute dry flexural strength and the absolute dry compressive strength of phosphorus building gypsum were compared under different mass ratios of material to ball, ball-milling speed, and ball-milling time, and the NSGM (1,4) model was applied to model and predict the strength of phosphorus building gypsum modified by ball milling. According to the research results, under the same mass ratio of material to ball and ball-milling speed, the absolute dry flexural strength and absolute dry compressive strength of phosphorus building gypsum firstly increased and then decreased with the increase in milling time. The NSGM (1,4) model established in this paper could effectively simulate and predict the absolute dry flexural strength and the absolute dry compressive strength of the ball-milling-modified phosphorus building gypsum; the average relative simulation errors were 12.38% and 13.77%, and the average relative prediction errors were 6.30% and 12.47%.

## 1. Introduction

Phosphogypsum is the industrial byproduct discharged from the wet preparation of phosphoric acid [1,2,3]. Its main component is CaSO_4_·2H_2_O, and it also contains other harmful impurities [4,5]. The phosphogypsum emissions in China are massive, and the pile stock has increased year by year. Currently, the pile stock amounts to 500 million tons, but the utilization rate is only about 15% [6]. The accumulation of phosphogypsum occupies a large amount of land and damages the natural environment [7]. Phosphorus building gypsum can be obtained through the dehydration of phosphogypsum at high temperatures, and its main component is CaSO_4_·1/2H_2_O [8]. The main application of phosphorus building gypsum is in preparing building materials, such as blocks and wall panels [9]. Blocks use phosphorus building gypsum as the main raw material, adding water before mixing, casting, molding, and drying to generate lightweight phosphorus building gypsum products. Blocks have many advantages, such as sound insulation, fire prevention, and convenient construction. They represent a new low-carbon, environmentally friendly, and healthy wall material. Wall panels also use phosphorus building gypsum as the main raw material, which can be mixed with a proper amount of fiber material as the core material before molding, cutting, drying, and other processes. Wall panels are mainly used for partitions, interior walls, etc. Phosphorus building gypsum can also be mixed with water and retarder into gypsum slurry, which can be used as indoor paint. The preparation cost of phosphorus building gypsum is low, which fits with the low-carbon development trend of the global building materials industry, and it has been recognized as one of the most promising methods for treating phosphogypsum [10].

However, due to its poor performance, phosphorus building gypsum cannot be applied widely in practical projects. On the one hand, the composition of phosphorus building gypsum is relatively complex, and the large number of impurities affects its performance. On the other hand, phosphorus building gypsum has poor crystal morphology, generally existing in a sheet or plate shape, which results in poor fluidity and high porosity. Therefore, the strength of phosphorus building gypsum is low, and the water resistance is poor [11]. It is necessary to apply some preprocessing methods in the preparation of phosphorus building gypsum to improve the performance of phosphogypsum, such as flotation methods [12], chemical methods [13], sieving methods [14], and heat treatment methods [15]. As a common powder preprocessing method, ball milling has been widely applied and studied in the preparation and modification of cement materials [16,17,18]. However, there have been few studies on the treatment of phosphogypsum and phosphorus building gypsum. Li [19] analyzed the influence of ball-milling time on the specific surface area, particle size distribution, 2 h strength, and absolute dry strength of phosphorus building gypsum at a standard consistency. It was found that the performance of phosphorus building gypsum was best when the ball-milling time was 3 min, and the increase in ball-milling time could not improve the performance of phosphorus building gypsum. Wu [20] studied the effects of ball-milling time on the particle size, physical properties, and mechanical properties of net slurry, and the results showed that the performance of phosphorus building gypsum could be enhanced by controlling the ball-milling time.

At present, artificial neural networks, polynomial regression analysis, machine learning, response surface fitting, and other methods are widely applied to model and predict the performance of building materials [21,22,23,24]. The prediction model of material performance can be used to guide the property design and optimization of building materials. However, research on the modification of phosphorus building gypsum using ball milling has focused on analyzing the change rules and mechanisms of material properties, whereas related studies on predicting the properties of phosphorus building gypsum using mathematical models are rare. This paper introduces gray theory to model and predict the strength of phosphorus building gypsum modified by ball milling. Gray theory can be applied to the analysis and prediction of systems with small data and uncertainty [25,26]. The gray prediction model has achieved good results in various fields, such as energy, economy, and environmental protection [27,28,29]. Furthermore, it has also been widely applied in the study of building material properties [30,31,32]. Zhou [33] applied the GM (1,4) model to complete the optimal design of low-shrinkage cementitious materials. Ma [34] used the GM (1,8) model to model and predict the 28-day compressive strength of calcium-based geopolymer based on pore structure characteristics and obtained good prediction results.

Through the experiments in this paper, the influence of ball milling with different parameters on the absolute dry flexural strength and absolute dry compressive strength of phosphorus building gypsum was investigated. Furthermore, as a function of the mass ratio of material to ball, ball-milling speed, and ball-milling time, the NSGM (1,4) model was adopted to model the absolute dry flexural strength and the absolute dry compressive strength of phosphorus building gypsum modified by ball milling, thereby realizing effective simulation and prediction.

## 2. Materials and Methods

### 2.1. Raw Materials

The phosphogypsum used was a light yellow powder obtained from a phosphogypsum yard of Yunnan Yuntianhua Co., Ltd. (Yunnan, China). Its chemical composition is shown in Table 1.

### 2.2. Experimental Design

The raw materials of phosphogypsum were dehydrated in an air-blowing thermostatic oven at 140 °C for 6 h to produce phosphorus building gypsum. Then, a small horizontal experimental ball mill was used for milling. The effects of different ratios of material to ball mass, milling speed, and milling time on the strength of phosphorus building gypsum were compared, as shown in Table 2.

### 2.3. Experimental Methods

According to the Chinese national standard “Gypsum Plasters—Determination of Mechanical Properties” (GB/T 17669.3-1999), the mechanical properties of phosphorus building gypsum after ball milling in each group were tested under water consumption for standard consistency.

## 3. Results and Discussion

### 3.1. The Effect of Ball Milling on Absolute Dry Flexural Strength of Phosphorus Building Gypsum

Table 3 shows the influence of different ball-milling parameters on the strength of phosphorus building gypsum.

**Figure 1:**
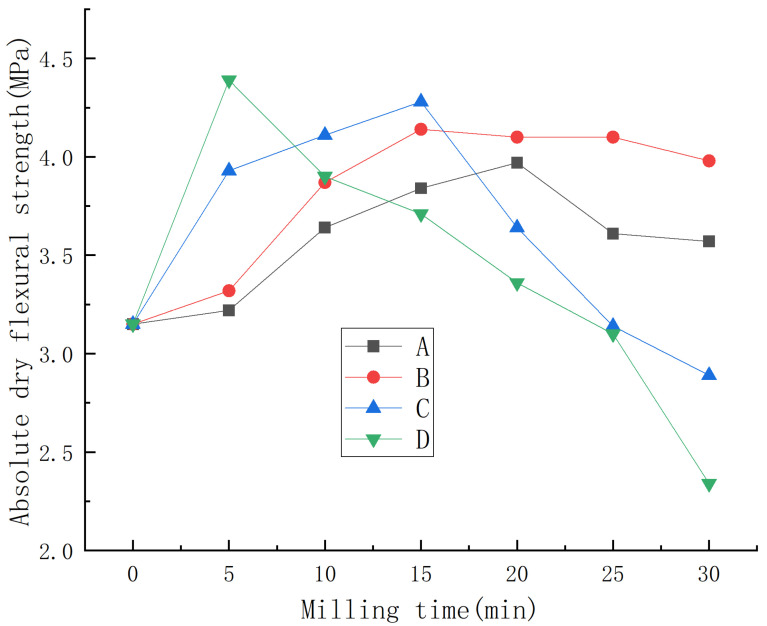
Effect of ball milling on absolute dry flexural strength of phosphorus building gypsum.

Under water consumption for standard consistency, the effects of the mass ratio of material to ball, milling speed, and milling time on the absolute dry flexural strength of phosphorus building gypsum are shown in Figure 1.

As can be seen from Figure 1, under different mass ratios of material to ball and milling speed, the absolute dry flexural strength of phosphorus building gypsum first increased and then decreased with the increase in milling time. When the mass ratio of material to ball was the same, faster ball milling had a greater influence on the absolute dry flexural strength of phosphorus building gypsum. When the milling speed was the same, a smaller mass ratio of material to ball had a greater influence on the absolute dry flexural strength of phosphorus building gypsum.

In general, under the conditions of a mass ratio of material to ball of 1:1, ball milling speed of 90 rpm, and ball milling time of 5 min, the absolute dry flexural strength of phosphorus building gypsum reached the maximum value of 4.39 MPa, increasing by 39% compared with the control group.

### 3.2. The Effect of Ball Milling on Absolute Dry Compressive Strength of Phosphorus Building Gypsum

**Figure 2:**
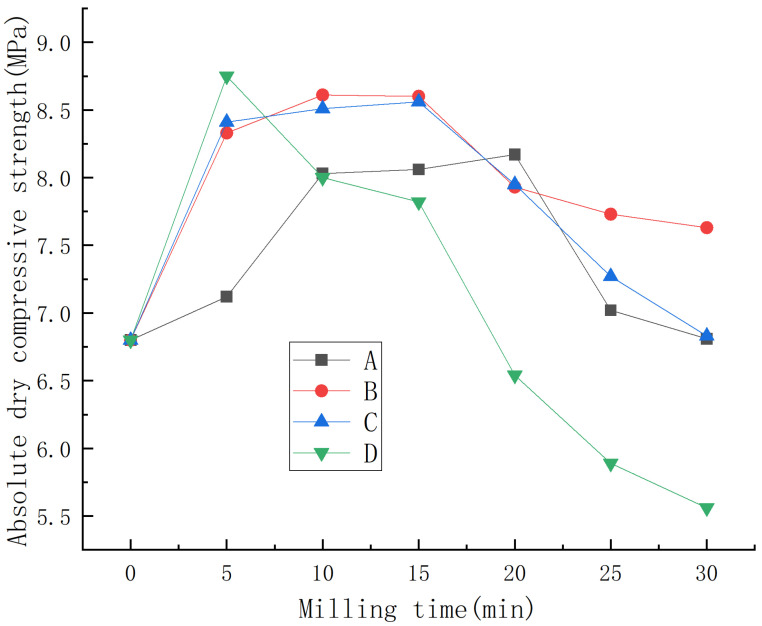
Effect of ball milling on absolute dry compressive strength of phosphorus building gypsum.

Under water consumption for standard consistency, the effects of the mass ratio of material to ball, milling speed, and milling time on the absolute dry compressive strength of phosphogypsum are shown in Figure 2.

As can be seen from Figure 2, under different mass ratios of material to ball and milling speed, the absolute dry compressive strength of phosphorus building gypsum first increased and then decreased with the increase in milling time. When the mass ratio of material to ball was the same, faster ball milling had a greater influence on the absolute dry compressive strength of phosphorus building gypsum. When the milling speed was the same, a smaller mass ratio of material to ball had a greater influence on the absolute dry compressive strength of phosphorus building gypsum.

In general, under the conditions of a mass ratio of material to ball of 1:1, ball milling speed of 90 rpm, and ball milling time of 5 min, the absolute dry compressive strength of phosphorus building gypsum reached the maximum value of 8.75 MPa, increasing by 29% compared with the control group.

### 3.3. Mechanism Analysis

By controlling the milling time, the rhomboid sheet crystal of phosphorus building gypsum could be changed to elliptic sheet crystal, and the relative thickness of phosphorus building gypsum crystal could be reduced. When the slurry was formed by adding water to phosphorus building gypsum, the phosphorus building gypsum crystal particles that were close to an oval shape with a relatively low thickness after ball milling had better fluidity. At the same time, the fine end particles of phosphorus building gypsum after ball milling increased, which changed the particle grading of phosphorus building gypsum, and the particles were effectively filled step by step, increasing the bulk density of phosphorus building gypsum after ball milling. Therefore, the overall structure of phosphorus building gypsum became denser, the porosity decreased, and the performance was improved.

However, when the ball milling time was too long, there were too many fine end particles of phosphorus building gypsum, and the specific surface area was too large, which increased the water consumption of standard consistency, thus adversely affecting the performance of phosphorus building gypsum.

## 4. NSGM (1,4) Prediction of Material Properties

### 4.1. Modeling

The NSGM (1,N) model was proposed by Professor Bo Zeng [25]. The difference model was applied to replace the shadow formula on the traditional GM (1,N) model to optimize the structure of the multivariable gray prediction model and improve the accuracy of prediction.

In this paper, the absolute dry flexural strength and absolute dry compressive strength were two indicators applied to investigate phosphorus building gypsum modified by ball milling, and there were two characteristic data in the system for modeling. Furthermore, three related parameters of the modeling system were investigated, namely the mass ratio of material to ball, the ball-milling speed, and the ball-milling time. The NSGM (1,4) model of absolute dry flexural strength-to-mass ratio of material to ball, ball-milling speed, and ball-milling time, and the NSGM (1,4) model of absolute dry compressive strength-to-mass ratio of material to ball, ball-milling speed, and ball-milling time were established. Below, we mainly describe the modeling process of absolute dry flexural strength, as the modeling process of absolute dry compressive strength is similar.

#### 4.1.1. Determination of the Absolute Dry Flexural Strength Modeling Data

The sample sequence involved 28 groups, and 23 groups of sample data were selected to construct the NSGM (1,4) model and test its simulation performance. Four groups of sample data were selected (mass ratio of material to ball of 2:1, milling speed of 70 rpm, and milling time of 30 min; mass ratio of material to ball of 2:1, milling speed of 90 rpm, and milling time of 30 min; mass ratio of material to ball of 1:1, milling speed of 70 rpm, and milling time of 30 min; mass ratio of material to ball of 1:1, milling speed of 90 rpm, and milling time of 30 min) to analyze the model effect, and the data are shown in Table 4.

The characteristic sequence of the constructed system was as follows:

$$\begin{array}{l} X_{1}^{\left(0\right)}=(3.15,3.22,3.64,3.84,3.97,3.61,3.15,3.32,3.87,4.14,4.1,4.1,\\ 3.15,3.93,4.11,4.28,3.64,3.14,\;3.15,4.39,3.9,3.71,3.36,2.4). \end{array}$$

The relevant behavior sequence was constructed as follows:

$$X_{2}^{\left(0\right)}=\left(2,2,2,2,2,2,2,2,2,2,2,2,1,1,1,1,1,1,1,1,1,1,1,1\right),$$

$$\begin{array}{l} X_{3}^{\left(0\right)}=(70,70,70,70,70,70,90,90,90,90,90,90,\\ 70,70,70,70,70,70,90,90,90,90,90,90), \end{array}$$

$$X_{4}^{\left(0\right)}=\left(0,5,10,15,20,25,0,5,10,15,20,25,0,5,10,15,20,25,0,5,10,15,20,25\right).$$

#### 4.1.2. Calculation of the NSGM (1,4) Model Parameters of Absolute Dry Flexural Strength

The accumulative sequences of system characteristics and related behavior were calculated to generate the following:

$$\begin{array}{l} X_{1}^{\left(1\right)}=(3.15,6.37,10.01,13.85,17.82,21.43,24.58,27.9,31.77,35.91,40.31,44.41,\\ 47.56,51.49,55.6,59.88,63.52,66.66,69.81,74.2,78.1,81.81,85.17,87.51), \end{array}$$

$$X_{2}^{\left(1\right)}=\left(2,4,6,8,10,12,14,16,18,20,22,24,25,26,27,28,29,30,31,32,33,34,35,36\right),$$

$$\begin{array}{l} X_{3}^{\left(1\right)}=(70,140,210,280,350,420,510,600,690,780,870,960,\\ 1030,1100,1170,1240,1310,1380,1470,1560,1650,1740,1830,1920), \end{array}$$

$$\begin{array}{l} X_{4}^{\left(1\right)}=(0,5,15,30,50,75,75,80,90,105,125,150,\\ 150,155,165,180,200,225,225,230,240,255,275,300). \end{array}$$

The mean sequence adjacent to the characteristic sequence of the system was calculated as follows:

$$\begin{array}{l} Z_{1}^{\left(1\right)}=(4.76,8.19,11.93,15.835,19.625,23.005,26.24,29.835,33.84,38.11,42.36,\\ 45.985,49.525,53.545,57.74,61.7,65.09,68.235,72.005,76.15,79.995,83.49,86.34). \end{array}$$

The parameter matrices B and Y of the NSGM (1,4) model were constructed, and the model parameters were calculated as follows:

$$\;\hat{p}={\left(b_{2},b_{3},b_{4},a,h_{1},h_{2}\right)}^{T}:$$

$$B=\left[\begin{array}{cccccc} 4 & 140 & 5 & -4.76 & 1 & 1\\ 6 & 210 & 15 & -8.19 & 2 & 1\\ 8 & 280 & 30 & -11.93 & 3 & 1\\ \ldots & \ldots & \ldots & \ldots & \ldots & \ldots \\ 36 & 1920 & 300 & -86.34 & 23 & 1 \end{array}\right],Y=\left[\begin{array}{c} 3.22\\ 3.64\\ 3.84\\ \ldots \\ 2.4 \end{array}\right].$$

The calculated model parameters were as follows:

$$\begin{array}{l} \;\hat{p}={\left(b_{2},b_{3},b_{4},a,h_{1},h_{2}\right)}^{T}={\left(B^{T}B\right)}^{-1}B^{T}Y\\ \;={\left(0.100249,0.001441,-0.023347,-0.201391,-0.721565,2.884889\right)}^{T}. \end{array}$$

#### 4.1.3. Construction of the NSGM (1,4) Model of Absolute Dry Flexural Strength

The model parameters were introduced into the NSGM (1,4) model to calculate the intermediate variables $\mu_{1}$, $\mu_{2}$, $\mu_{3}$, and $\mu_{4}$:

$$\mu_{1}=\frac{1}{1+0.5a}=1.1119,$$

$$\mu_{2}=\frac{1-0.5a}{1+0.5a}=1.2320,$$

$$\mu_{3}=\frac{h_{1}}{1+0.5a}=-0.8024,$$

$$\mu_{4}=\frac{h_{2}-h_{1}}{1+0.5a}=4.0103.$$

Furthermore, the time response of the NSGM (1,4) model of absolute dry flexural strength of ball-milling-modified phosphorus building gypsum could be obtained as follows:

$${\;\hat{X}}_{1}^{\left(1\right)}\left(k\right)=\overset{k-1}{\underset{t=1}{\sum }}\left[\overset{N}{\underset{i=2}{\sum }}1.1119{\left(1.2320\right)}^{t-1}b_{i}X_{i}^{\left(1\right)}\left(k-t+1\right)\right]+{\left(1.2320\right)}^{k-1}{\;\hat{X}}_{1}^{\left(1\right)}\left(1\right)\;+\overset{k-2}{\underset{j=0}{\sum }}{\left(1.2320\right)}^{j}\left[-0.8024\left(k-j\right)+4.0103\right],\;k=1,2,3,\ldots$$

The incremental formula of the NSGM (1,4) model for the absolute dry flexural strength of phosphorus building gypsum modified by ball milling was as follows:

$${\;\hat{X}}_{1}^{\left(0\right)}\left(k\right)={\;\hat{X}}_{1}^{\left(1\right)}\left(k\right)-{\;\hat{X}}_{1}^{\left(1\right)}\left(k-1\right),\;k=2,3,4,\ldots$$

#### 4.1.4. NSGM (1,4) Model of Absolute Dry Compressive Strength

The modeling process was the same as the above steps, and the time response of the NSGM (1,4) model for the absolute dry compressive strength of phosphorus building gypsum modified by ball milling was obtained as follows:

$${\;\hat{X}}_{1}^{\left(1\right)}\left(k\right)=\overset{k-1}{\underset{t=1}{\sum }}\left[\overset{N}{\underset{i=2}{\sum }}1.1271{\left(1.2542\right)}^{t-1}b_{i}X_{i}^{\left(1\right)}\left(k-t+1\right)\right]+{\left(1.2542\right)}^{k-1}{\;\hat{X}}_{1}^{\left(1\right)}\left(1\right)\;+\overset{k-2}{\underset{j=0}{\sum }}{\left(1.2542\right)}^{j}\left[-0.7731\left(k-j\right)+8.1241\right],\;k=1,2,3,\ldots$$

### 4.2. Model Simulation, Prediction Results, and Test Errors

#### 4.2.1. Test of Absolute Dry Flexural Strength Model of Phosphorus Building Gypsum Modified by Ball Milling

**Figure 3:**
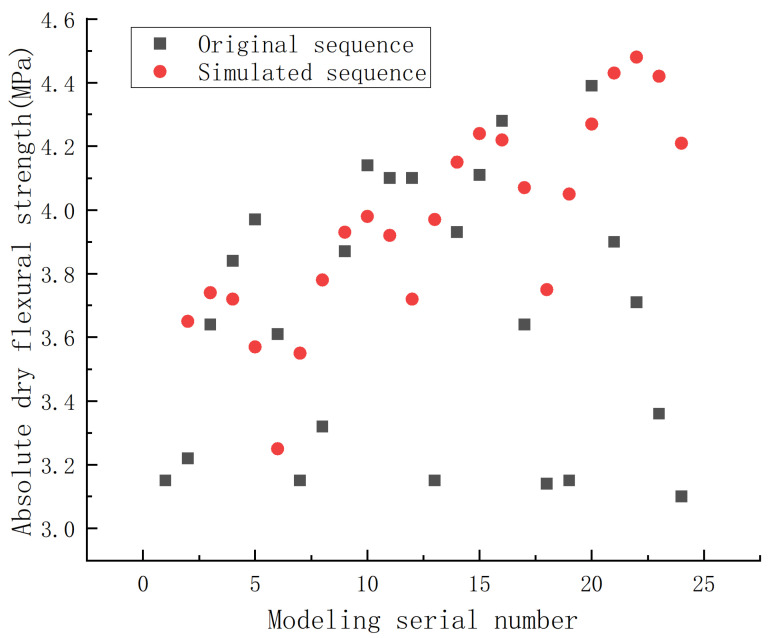
Comparison of the original sequence and the simulated sequence in absolute dry flexural strength of the phosphorus building gypsum modified by ball milling.

Using the established NSGM (1,4) model, the simulated value of absolute dry flexural strength of phosphorus building gypsum modified by ball milling could be calculated, and the result is shown in Figure 3.

The residual error was calculated as follows:

$$\varepsilon_{S}\left(k\right)=X_{1}^{\left(0\right)}\left(k\right)-{\;\hat{X}}_{1}^{\left(0\right)}\left(K-1\right),k=2,3,4,\ldots$$

The relative simulation error was calculated as follows:

$$\Delta_{S}\left(k\right)=\left|\frac{\varepsilon_{S}\left(k\right)}{X_{1}^{\left(0\right)}\left(k\right)}\times 100\%\right|,k=2,3,4,\ldots$$

The average relative simulation error was calculated as follows:

$$\bar{\Delta_{S}}=\frac{1}{n}\overset{n}{\underset{k=1}{\sum }}\Delta_{S}\left(k\right).$$

The average relative simulation error of the calculated NSGM (1,4) model of absolute dry flexural strength was 12.38%, as shown in Table 5.

From the error level reference table of the grey prediction test model, it can be seen that the prediction accuracy level of the model was level 1, which means that the model could be applied to predict the absolute dry flexural strength of phosphorus building gypsum as a function of the mass ratio of material to ball, ball milling speed, and ball milling time. The prediction results and errors are shown in Table 6.

Table 6 shows that the average relative prediction error of the absolute dry flexural strength of ball-milling-modified phosphorus building gypsum predicted by the NSGM (1,4) model was 6.30%. Although there was some error, this model could be applied to the performance prediction of phosphorus building gypsum modified by ball milling in engineering.

#### 4.2.2. Test of Absolute Dry Compressive Strength Model of Phosphorus Building Gypsum Modified by Ball Milling

**Figure 4:**
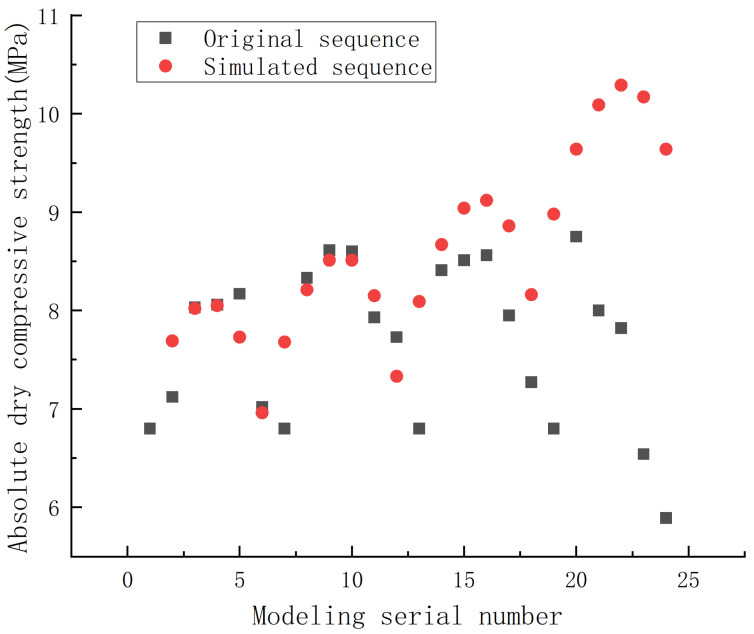
Comparison of the original sequence and the simulated sequence in absolute dry compressive strength of the phosphorus building gypsum modified by ball milling.

Using the established NSGM (1,4) model, the simulated value of absolute dry compressive strength of phosphorus building gypsum modified by ball milling could be calculated, and the result is shown in Figure 4.

The average relative simulation error of the calculated NSGM (1,4) model of absolute dry compressive strength was 13.77%, as shown in Table 7.

From the error-level reference table of the gray prediction test model, it can be seen that the prediction accuracy level of the model was level 1, which means that the model could be applied to predict the absolute dry compressive strength of phosphorus building gypsum as a function of the mass ratio of material to ball, ball-milling speed, and ball-milling time. The prediction results and errors are shown in Table 8.

Table 8 shows that the average relative prediction error of absolute dry compressive strength of ball-milling-modified phosphorus building gypsum predicted by the NSGM (1,4) model was 12.47%. Although there was some error, this model could be applied to the performance prediction of phosphorus building gypsum modified by ball milling in engineering.

## 5. Potential Applications and Prospects

Because of the complex composition of phosphorus building gypsum, a large number of impurities affect its performance. Furthermore, the particle shape and particle size distribution of phosphorus building gypsum are not good, adversely affecting its performance. According to the results of this paper, it can be seen that after ball milling, various properties of the phosphorus building gypsum were improved, thus meeting the application requirements for building gypsum. Compared with other materials, the ball-milled-modified phosphorus building gypsum has certain advantages, as shown in Table 9, including simplicity, environmental friendliness, and low cost, as well as certain practical significance in production.

## 6. Conclusions

1. Different ball-milling parameters had a great influence on the mechanical properties of phosphorus building gypsum. Under the same mass ratio of material to ball and milling speed, the absolute dry flexural strength and compressive strength of phosphorus building gypsum increased first and then decreased with the increase in milling time. Under the same milling time, a smaller mass ratio of material to ball and a higher milling speed had a greater influence on the performance of phosphorus building gypsum.
2. Under the conditions of a mass ratio of material to ball of 1:1, milling speed of 90 rpm, and milling time of 5 min, the absolute dry flexural strength of phosphorus building gypsum reached the maximum value of 4.39 MPa, increasing by 39% compared with the control group. The absolute dry compressive strength reached the maximum value of 8.75 MPa, increasing by 29% compared with the control group.
3. The NSGM (1,4) model established in this paper could be used to conduct a valid simulation and prediction of the absolute dry flexural strength and compressive strength of phosphorus building gypsum modified by ball milling. The average relative simulation errors were 12.38% and 13.77%, and the average relative prediction errors were 6.30% and 12.47%.

## Figures and Tables

**Table 1:** Chemical content analysis of phosphogypsum.

Component	SiO_2_	Al_2_O_3_	Fe_2_O_3_	MnO	MgO	CaO	K_2_O	P_2_O_5_	SO_3_	Organisms
Content (%)	14.52	1.66	0.15	0.005	0.17	31.94	0.22	0.94	45.38	0.25

**Table 2:** Ball-milling test scheme.

No.	Mass Ratio of Material to Ball	Milling Speed (rpm)	Milling Time (min)
A0	2:1	70	0
A1	2:1	70	5
A2	2:1	70	10
A3	2:1	70	15
A4	2:1	70	20
A5	2:1	70	25
A6	2:1	70	30
B0	2:1	90	0
B1	2:1	90	5
B2	2:1	90	10
B3	2:1	90	15
B4	2:1	90	20
B5	2:1	90	25
B6	2:1	90	30
C0	1:1	70	0
C1	1:1	70	5
C2	1:1	70	10
C3	1:1	70	15
C4	1:1	70	20
C5	1:1	70	25
C6	1:1	70	30
D0	1:1	90	0
D1	1:1	90	5
D2	1:1	90	10
D3	1:1	90	15
D4	1:1	90	20
D5	1:1	90	25
D6	1:1	90	30

**Table 3:** Effects of different ball-milling parameters on the strength of phosphorus building gypsum.

No.	Absolute Dry Flexural Strength (MPa)	Absolute Dry Compressive Strength (MPa)
A0	3.15	6.8
A1	3.22	7.12
A2	3.64	8.03
A3	3.84	8.06
A4	3.97	8.17
A5	3.61	7.02
A6	3.57	6.81
B0	3.15	6.8
B1	3.32	8.33
B2	3.87	8.61
B3	4.14	8.6
B4	4.1	7.93
B5	4.1	7.73
B6	3.98	7.63
C0	3.15	6.8
C1	3.93	8.41
C2	4.11	8.51
C3	4.28	8.56
C4	3.64	7.95
C5	3.14	7.27
C6	2.89	6.83
D0	3.15	6.8
D1	4.39	8.75
D2	3.9	8
D3	3.71	7.82
D4	3.36	6.54
D5	3.1	5.89
D6	2.34	5.56

**Table 4:** Modeling data.

Test Serial Number	Absolute Dry Flexural Strength (MPa)	Absolute Dry Compressive Strength (MPa)	Modeling Serial Number
A0	3.15	6.8	1
A1	3.22	7.12	2
A2	3.64	8.03	3
A3	3.84	8.06	4
A4	3.97	8.17	5
A5	3.61	7.02	6
A6	3.57	6.81	25 (forecasting group)
B0	3.15	6.8	7
B1	3.32	8.33	8
B2	3.87	8.61	9
B3	4.14	8.6	10
B4	4.1	7.93	11
B5	4.1	7.73	12
B6	3.98	7.63	26 (forecasting group)
C0	3.15	6.8	13
C1	3.93	8.41	14
C2	4.11	8.51	15
C3	4.28	8.56	16
C4	3.64	7.95	17
C5	3.14	7.27	18
C6	2.89	6.83	27 (forecasting group)
D0	3.15	6.8	19
D1	4.39	8.75	20
D2	3.9	8	21
D3	3.71	7.82	22
D4	3.36	6.54	23
D5	3.1	5.89	24
D6	2.34	5.56	28 (forecasting group)

**Table 5:** Error of absolute dry flexural strength simulation results.

Modeling Serial Number	Original Sequence	Simulated Sequence	Relative Simulation Error (%)
2	3.22	3.65	13.39
3	3.64	3.74	2.81
4	3.84	3.72	3.03
5	3.97	3.57	10.05
6	3.61	3.25	9.85
7	3.15	3.55	12.63
8	3.32	3.78	13.78
9	3.87	3.93	1.52
10	4.14	3.98	3.77
11	4.1	3.92	4.35
12	4.1	3.72	9.38
13	3.15	3.97	25.99
14	3.93	4.15	5.57
15	4.11	4.24	3.16
16	4.28	4.22	1.37
17	3.64	4.07	11.77
18	3.14	3.75	19.49
19	3.15	4.05	28.43
20	4.39	4.27	2.62
21	3.9	4.43	13.49
22	3.71	4.48	20.78
23	3.36	4.42	31.51
24	3.1	4.21	35.89
Average relative simulation error = 12.38%			

**Table 6:** The prediction results of absolute dry flexural strength.

Modeling Serial Number	Original Sequence	Simulated Sequence	Relative Simulation Error (%)
25	3.57	3.91	9.52
26	3.98	3.54	11.08
27	2.89	3.01	4.02
28	2.34	2.35	0.60
Average relative prediction error = 6.30%			

**Table 7:** Error of absolute dry compressive strength simulation results.

Modeling Serial Number	Original Sequence	Simulated Sequence	Relative Simulation Error (%)
2	7.12	7.69	8.07
3	8.03	8.02	0.14
4	8.06	8.05	0.06
5	8.17	7.73	5.37
6	7.02	6.96	0.91
7	6.8	7.68	12.88
8	8.33	8.21	1.45
9	8.61	8.51	1.18
10	8.6	8.51	0.99
11	7.93	8.15	2.82
12	7.73	7.33	5.16
13	6.8	8.09	18.99
14	8.41	8.67	3.15
15	8.51	9.04	6.20
16	8.56	9.12	6.58
17	7.95	8.86	11.46
18	7.27	8.16	12.29
19	6.8	8.98	32.06
20	8.75	9.64	10.12
21	8	10.09	26.10
22	7.82	10.29	31.54
23	6.54	10.17	55.43
24	5.89	9.64	63.75
Average relative simulation error = 13.77%			

**Table 8:** The prediction results of absolute dry compressive strength.

Modeling Serial Number	Original Sequence	Simulated Sequence	Relative Simulation Error (%)
25	6.81	8.99	31.97
26	7.63	8.16	6.97
27	6.83	6.76	0.99
28	5.56	5.01	9.94
Average relative prediction error = 12.47%			

**Table 9:** Comparison of ball-milling-modified phosphorus building gypsum and other gypsum materials.

Performance Index	Untreated Phosphorus Building Gypsum	Ball-Milling-Modified Phosphorus Building Gypsum	Desulfurized Gypsum	Building Gypsum (GB/T 9775-2008) (Level 1)
Absolute dry flexural strength (MPa)	3.15	4.39	3.0	≥3.0
Absolute dry compressive strength (MPa)	6.8	8.75	6.4	≥5.0

## Data Availability

The data supporting this study’s findings are available on request from the corresponding author.
